# Flk1+ and VE-Cadherin+ Endothelial Cells Derived from iPSCs Recapitulates Vascular Development during Differentiation and Display Similar Angiogenic Potential as ESC-Derived Cells

**DOI:** 10.1371/journal.pone.0085549

**Published:** 2013-12-30

**Authors:** Erin E. Kohler, Kishore K. Wary, Fei Li, Ishita Chatterjee, Norifumi Urao, Peter T. Toth, Masuko Ushio-Fukai, Jalees Rehman, Changwon Park, Asrar B. Malik

**Affiliations:** 1 Department of Pharmacology and Center for Lung and Vascular Biology, The University of Illinois, Chicago, Illinois, United States of America; 2 Research Resources Center, The University of Illinois, Chicago, Illinois, United States of America; 3 Section of Cardiology, Department of Medicine, The University of Illinois, Chicago, Illinois, United States of America; Northwestern University, United States of America

## Abstract

**Rationale:**

Induced pluripotent stem (iPS) cells have emerged as a source of potentially unlimited supply of autologous endothelial cells (ECs) for vascularization. However, the regenerative function of these cells relative to adult ECs and ECs derived from embryonic stem (ES) cells is unknown. The objective was to define the differentiation characteristics and vascularization potential of Fetal liver kinase (Flk)1^+^ and Vascular Endothelial (VE)-cadherin^+^ ECs derived identically from mouse (m)ES and miPS cells.

**Methods and Results:**

Naive mES and miPS cells cultured in type IV collagen (IV Col) in defined media for 5 days induced the formation of adherent cell populations, which demonstrated similar expression of Flk1 and VE-cadherin and the emergence of EC progenies. FACS purification resulted in 100% Flk1^+^ VE-cadherin^+^ cells from both mES and miPS cells. Emergence of Flk1^+^VE-cadherin^+^ cells entailed expression of the vascular developmental transcription factor *Er71*, which bound identically to *Flk1, VE-cadherin*, and *CD31* promoters in both populations. Immunostaining with anti-VE-cadherin and anti-CD31 antibodies and microscopy demonstrated the endothelial nature of these cells. Each cell population (unlike mature ECs) organized into well-developed vascular structures *in* *vitro* and incorporated into CD31^+^ neovessels in matrigel plugs implanted in nude mice *in* *vivo*.

**Conclusion:**

Thus, iPS cell-derived Flk1^+^VE-cadherin^+^ cells expressing the Er71 are as angiogenic as mES cell-derived cells and incorporate into CD31^+^ neovessels. Their vessel forming capacity highlights the potential of autologous iPS cells-derived EC progeny for therapeutic angiogenesis.

## Introduction

The transduction of fibroblast cells with transcription factors *Nanog*, *Sox2*, *Oct4*, *Klf4*, and *c-Myc* converts these cells into induced pluripotent stem (iPS) cells [1-3]. The observations that adult mice can be derived from iPS cells indicate that these reprogrammed cells acquire embryonic stem (ES) cell-like properties, and therefore have the potential to generate any tissue [4,5]. An important aim of regenerative cell therapy is to use the iPS cells because they not only self-renew and have the potential to differentiate into mature cells [6,7], but because unlike ES cells, iPS cells can give rise to autologous cells that are ideal for personalized regenerative therapies [8,9]. 

During embryogenesis, primitive vascular ECs, termed angioblasts, and hematopoietic stem cells emerge from the mesodermal compartment in successive waves to form blood vessels [12-17]. The upstream components that induce exit of mesodermal cells to vascular cell progenies include factors such as bone morphogenetic proteins (BMPs), hypoxia, and Wnts [17-20]. A major subset of mesodermal cells expressing Flk1^+^Flt1^+^VE-cadherin^+^CD34^+^CD31^+^ are capable of forming vascular plexus-like structures [20-25]. Several studies have identified Flk-1 as an earliest marker of mesodermal stem cells and angioblasts [12,17,18,21]. In mice, Flk1+ cells differentiated into ECs to form primitive vascular structures through the process of vasculogenesis [12,15,17,18,21]. Binding to vascular endothelial growth factor (VEGF) to Flk1/VEGFR-2 regulates multiple aspects of neovascularization including EC development, survival, differentiation, migration, and lumenization [14,17,19-21]. The one-pass transmembrane protein VE-cadherin, which mediates cell-cell adhesion and contributes to the formation of adherens junctions (AJs), is expressed in both immature and mature ECs [20,21,23]. Analysis of the endothelial promoter/enhancer revealed the presence of ETS (E-twenty six) binding site that directly regulated expression of most, if not all, endothelial genes [26-33]. The transcription factors *Er71* (also known as *Etv2/Etsrp*), *FoxC2, Erg1* and *Fli1* were shown to regulate the development of vascular ECs [12,26-33]. Thus, the development of ECs entails timely expression and function of above key proteins.

In adults, there is only a limited pool of endothelial progenitor cells (EPCs) that contribute to neovascularization and repair *in situ* [8-12], and these EPCs are often dysfunctional or lost in patients with cardiovascular risk factors [10,11,12,34]. Although ECs have been isolated from mouse embryonic stem (mES) and human embryonic stem (hES) cells [35-41], it is unclear whether iPS cells can be used as a source of reparative ECs to induce revascularization. It is also not known whether miPS and mES cell-derived ECs have similar pattern of differentiation and function similarly to induce vascularization. Here we demonstrate the angiogenic potential of mES cell-derived ECs *vis-à-vis* iPS cell-derived ECs and show that Flk1^+^VE-cadherin^+^ cells generated from either stem cells integrated into CD31^+^ neovessels *in vivo*, and that vascular differentiation of iPS cells involves the developmental transcription factor Er71, thus suggesting that iPS differentiation mirrors the differentiation seen in ES cells and during physiological embryonic development. 

## Materials and Methods

### Reagents

The mouse induced Pluripotent Stem (iPS) cells (iMZ-9 and iMZ-21) were a kind gift from Dr. Kristin K. Baldwin (The Scripps Research Institute, La Jolla, CA) [4,5]. The undifferentiated mouse embryonic stem cells (mES cells, J1 line) were purchased from American Type Culture Collection (Manassas, VA). The rat anti-mouse CD31 antibody (IgG_2a_) (BD 550274) was purchased from BD Biosciences/Pharmingen (San Jose, CA). The anti-Sp1 antibody (ab13370) was bought from Abcam (Cambridge, MA). The anti-Etv2/Er71 (W-14) was purchased from Santa Cruz (Santa Cruz, CA). The goat anti-Rat IgG2α FITC (#A110-109F) was purchased from Bethyl Laboratories (Montgomery, TX).

### Cell Culture

The mouse ESC line (J1), and iPS cells iMZ-9 and iMZ-21 [4,5] were propagated and maintained using mitomycin-blocked mouse embryonic fibroblast cells (MEF) and Leukemia Inhibitory Factor (LIF; Chemicon/Millipore, Billerica, MA). Briefly, mESCs or iPSCs were cultured in the ES cell medium consisting of high glucose-Dulbecco's Modified Eagles medium (Invitrogen, Carlsbad, CA) supplemented with 15% ES-qualified fetal bovine serum (FBS; Invitrogen), 2 mM L-glutamine (Invitrogen), 1 mM sodium pyruvate, 0.1 mM nonessential amino acids (Invitrogen), penicillin (10 µg/ml), streptomycin (5 µg/ml), 0.1 mM β-mercaptoethanol (Invitrogen), and 500 U/ml recombinant LIF (ESGRO®; Millipore) at 37°C with 5% CO_2_. The cells were passed every 3 to 4 days at a ratio of 1:15 using a 1 mM EDTA PBS solution as the dissociation buffer. For the differentiation studies, mESC or iPSC cells were passed twice in gelatin-coated culture dishes at a 1:3 dilution without the presence of MEF feeders. To initiate differentiation, the MEF-free pre-conditioned cells were dissociated with 1 mM EDTA PBS and seeded on IV Col-coated dishes (BD Bioscience, San Jose, CA) at a density of 35,000/35 mm dishes in serum-free differentiation medium consisting of 75% IMDM, 25% Ham’s F12 medium, N-2 and B-27 Supplements (without Vitamin A), 0.05% BSA (all from Gibco), 4.5x10e-4 M 1-thioglycerol (MTG) and 0.5 mM ascorbic acid (both from Sigma, St Louis, MO). The serum-free medium was supplemented with human BMP-4 at 2 ng/ml (R&D Systems), human VEGF^165^ at 50 ng/ml (Miltenyi Biotec), and human basic FGF at 10 ng/ml (Millipore). 

### Flow Cytometric Analysis and Sorting for Subcultures

Differentiated mES and iPS cells were collected following dissociation with a 1 mM EDTA PBS solution. Single cell suspensions were prepared and labeled with goat-anti-mouse VE-cadherin (R&D Systems, Minneapolis, MN) and donkey anti-goat secondary antibody coupled with Alexa Fluor 488 (AF-488) (eBioscience, San Diego, CA). The other primary antibodies used for the analysis or sorting included rat anti-mouse Flk1 (clone avas 12a) coupled with allophycocyanin (APC) (eBioscience) and rat-anti-mouse CD41 coupled with R-phycoerythrin (PE) (BD Bioscience). Immuno-fluorescence-labeled cell populations bearing specific marker(s) were analyzed on a CyAn ADP Analyzer (Beckman Coulter) or sorted using a Moflo high speed sorter (Becton Dickinson, Franklin Lakes, NJ). To test the lineage characteristics and colony formation of the sorted cells, the cells were subcultured at 50,000 per 35 mm type IV collagen Biocoat dishes with the serum-free differentiation medium. The number and identities of endothelial and hematopoietic colonies were counted and confirmed *in situ* using goat anti-mouse VE-cadherin (R&D Systems, Minneapolis, MN) and donkey anti-goat secondary antibody coupled with Alexa Fluor 488 (AF-488) (eBioscience) as well as rat-anti-mouse CD41 coupled with R-phycoerythrin (PE) (BD Biosciences) for the early hematopoietic lineages. 

### Gene Expression Analysis

The profile of pluripotent, mesoderm, hemangioblast, angioblast, hematopoietic and mature EC markers were quantified using quantitative (q) RT-PCR as previously described by us [42,43]. Q-RT-PCR assays were performed using the ABI Prism 7700 Sequence Detection System (Applied Biosystems, Carlsbad, CA) according to the manufacturer's instructions. For oligonucleotide information, please see Table 1. The experiments were carried out 5 times at least in triplicate for each gene target.

**Table 1:** Mouse quantitative RT-PCR primers.

**Genes**	**Sequences**	**Product Size**	**Accession #**
*c-Myc*	For 3’-*ACAAGCTCACCTCTGAAAAGGACT*-5’ For 3’-*CTCGAGTTAGGTCAGTTTATGCAC*-5’	105bp	L00038.1
*Oct4*	For 3’-*CCACTTCACCACACTCTACTCAG*-5’ Rev 3’-*AAGCTCCAGGTTCTCTTGTCTAC*-5’	165 bp	NM_013633
*Gapdh*	For 3’-*GACAATGAATACGGCTACAGCA*-5’ Rev 3’-*GTTATTATGGGGGTCTGGGATG*-5’	182 bp	GU214026
*Klf4*	For 3’-*GAGGAAGCGATTCAGGTACAGAAC*-5’ Rev 3’-*AGGCTTATTTACCTGGCTTAGGTC*-5’	182 bp	NM_010637
*Sox2*	For 3’-*CTAGTGGTACGTTAGGCGCTTC*-5’ Rev 3’-*GCCCGGAGTCTAGCTCTAAATA*-5’	73 bp	U31967.1
*Brachyury*	For 3’-*CATTACACACCACTGACACACAC*-5’ Rev 3’-*AGTCTCAGCACATGGAGGAAAGT*-5’	195 bp	BC120807
*Er71*	For 3’-*GACTACACCACCACGTGGAATAC*-5’ Rev 3’-*AGACTGCTTGTTCGATTTGGAG*-5’	104 bp	L10427
*Flk1*	For 3’-*TGTGGTCTCACTACCAGTTAAAGC*-5’ Rev 3’-*CATTCGATCCAGTTTCACAGAG*-5’	95 bp	NM_010612.2
*VE-cadherin*	For 3’-*AGATCCCAGAAGAGCTAAGAGGAC*-5’ Rev 3’-*AGAAAAGGAAGAGTGAGTGACCAG*-5’	98 bp	NM_009868.4
*CD31*	For 3’-*GAGACTCAGAGGCGCTAGTTAAT*-5’ Rev 3’-*CTAACCCAGTGATTGACAACAGA*-5’	195 bp	NM_008816.2

### Chromatin Immunoprecipitation (ChIP) Assay

ChIP experiments have been previously described [42,44]. ChIP kit was purchased from Thermo Fisher Scientific (Rockford, IL). The chromatins were pre-cleared and then subjected to immunoprecipitation with specific antibodies. For control, anti-Glut-1 and anti-Sp-1 were used. The immunoprecipitated DNAs were analyzed by PCR using primer pairs that amplify the region of the *Flk1*-, *VE-cadherin*-, and *CD31*-promoter/enhancer DNA sequences.

### Matrigel Assay, Hind limb ischemia, Immunofluorescence and Microscopy

Three-month-old athymic nude mice (Harlan Laboratory, Madison, WI) were used for this assay. All experiments involving nude and C57 mice reported in this study were carried-out under the Institutional Animal Care and Use Committee (IACUC) Animal Welfare Assurance No. A3460-01 and UIC ACC#13-069. Experiments were conducted according to IACUC and NIH guidelines. The mice were housed in the University of Illinois at Chicago Animal Care Vivarium under pathogen-free conditions and treated according to the UIC protocol for Animal Care Committee (ACC). For post-surgical pain managements buprenex was used. The mouse ES cell line (J1) and iPS (iMZ21) cell line derived Flk1^+^/VE-cadherin^+^ cells were incubated in lentivirus encoding *mCherry* (BioVision, Mountain View, CA) in media containing mCherry (~10^7^ particles/ml) overnight in complete differentiation media as previously described [42,43]. Growth factor-reduced 200 µL Matrigel (BD Biosciences, San Jose, CA) + 2 million mCherry-treated cells + 30 µl VEGF^165^ (Lonza [Walkersville, MD] #CC-3202) + Wnt3a (2 ng/µl) (R&D Systems) were injected subcutaneously into the midventral abdomen of the mice. Plugs were allowed to solidify, and the mice were monitored every 24 hours to assess the wound. After 7 days, the plugs were retrieved, washed in PBS, and fixed with 4% PFA. Five-micrometer serial sections were prepared (University of Illinois at Chicago Research Histology and Tissue Imaging Core, Chicago) and stained with hematoxylin and eosin (H&E). Femoral artery ligation to induce hind limb ischemia in C57 mice have been described previously [45]. Immunofluorescent staining, microscopy, and quantification have been previously described [42-44]. For quantification, 10-12 microscopic fields were randomly selected, with 10X or 20X magnifications in 6 different sections. A digital camera was used to capture images, which were then saved as TIFF documents. Composite figures were assembled and labeled with the use of QuarkXpress 8.1.2 software (Quark Inc., Denver, CO), and then the images were converted into the EPS document.

### Statistical Analysis

GraphPad Prizm 5.0 (GraphPad Software, Inc., La Jolla, CA) was used to analyze data as described previously [44,45]. The data represent mean±S.E.M. Analysis of variance (ANOVA) was performed with posthoc comparisons using an unpaired *t* test or Mann–Whitney tests, as appropriate. P<0.05 is considered significant.

## Results

### Differentiation of mES and miPS cells into Flk1^+^VE-cadherin^+^ cells

**Figure 1:**
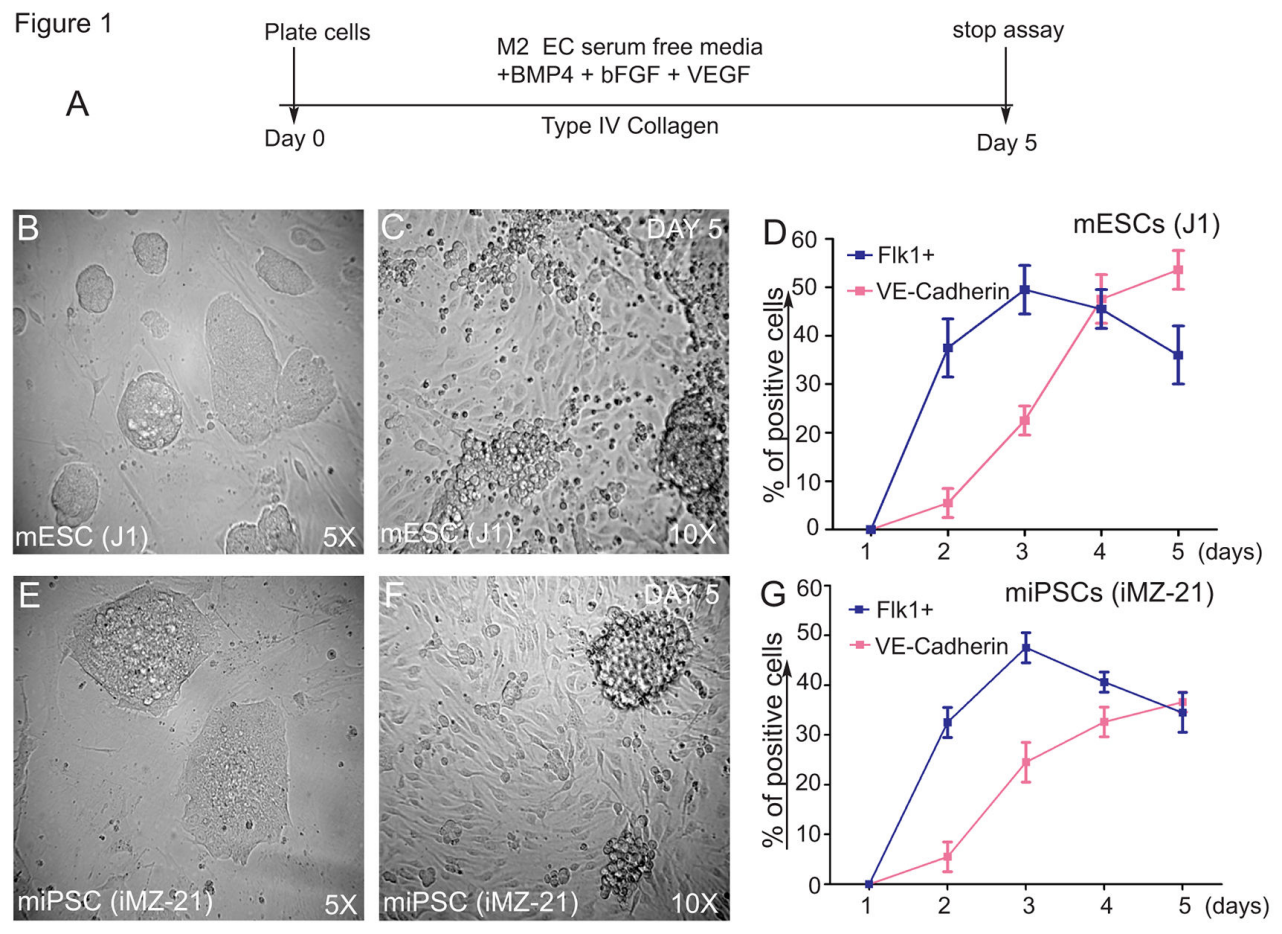
Induction of Flk1^+^VE-cadherin^+^ vascular EC progenies from iPS and ES cells. Timeline of emergence of Flk1^+^VE-cadherin^+^ vascular ECs (**A**). Undifferentiated mES (J1 line) or miPS (iMZ-21) cells were cultured for 5 d in IV Col-coated dishes in media containing BMP4, bFGF, and VEGF^165^ to induce generation of vascular EC progenies. (B&C) Representative phase contrast microscopy of mES cell-derived adherent vascular progenies after d 5 in culture at indicated magnifications (B&C). Representative phase contrast microscopy of miPS cell-derived vascular progenies after day 5 in culture at the indicated magnifications (E&F). FACS profile of the emergence of Flk1^+^VE-cadherin^+^ vascular progenies (D&G). All experiments were repeated >5 times. Data indicate the mean±S.E.M. n=5.

Since mesodermal-ECs are anchorage-dependent cells known to adhere onto IV Col, we used IV Col as the supporting matrix protein in these studies. The experimental timeline and differentiation conditions are as shown (Figure 1A). To induce formation of Flk1^+^ vascular progenies, media containing LIF was removed from naive mES and miPS cells, and the cells were cultured on IV Col-coated dishes and propagated in presence of complete media containing BMP4, VEGF, and bFGF. Using mouse ES (J1 line) and iPS (iMZ-21 line) cells in this culture condition, we observed the emergence of adherent cells on day 5 (Figure 1B&C, E&F). The appearance of Flk1^+^ or VE-cadherin^+^ cells was monitored using anti-mouse-Flk1 and anti-mouse-VE-cadherin mAbs and the cells were sorted using FACS. mES cells gave rise to 38+5% Flk1^+^ cells on day 2 and peaked at 49+7% on day 3, whereas these numbers dropped to 46+5% and 37+6% on days 4 and 5, respectively. The yield of VE-cadherin^+^ cells from mES was 7+3% on day 2 and increased steadily to 23+3%, 47+5% and 52+4% on days 3, 4 and 5, respectively (Figure 1D). The emergence of Flk1^+^ and VE-cadherin^+^ cells from miPS cells followed similar trends. Percentage of Flk1^+^ cells derived from iPS cells were 32+3%, 48+4%, 42+3%, 35+3% on days 2, 3, 4 and 5, respectively (Figure 1G); while the yield of VE-cadherin^+^ cells from miPS was 6+3% on day 2 and increased steadily to 25+3%, 32+3% and 37+2% on days 3, 4 and 5, respectively. The co-expression of Flk1 and VE-cadherin was used as an indicator of true vascular differentiation. Hematopoietic clusters made of round cells were not quantified. 

**Figure 2:**
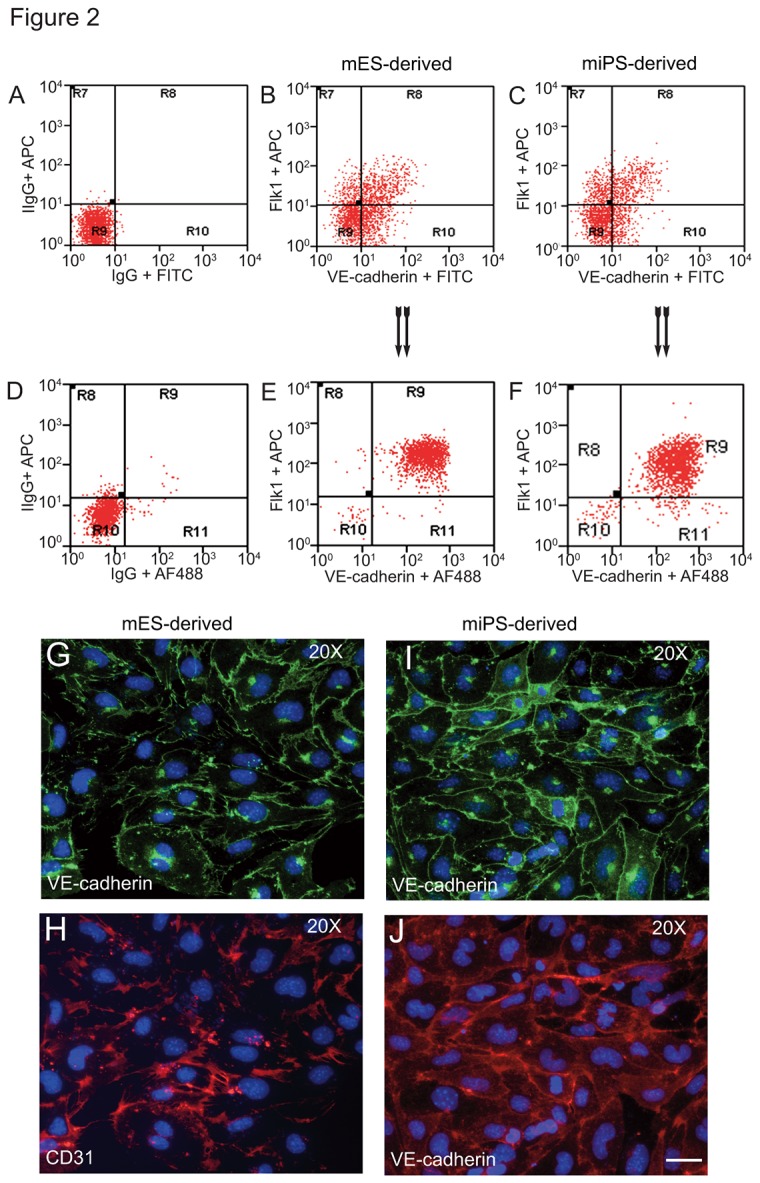
FACS analysis of emerging vascular EC progenies from mES and iPS cells. Adherent cells (2x10^5^) were detached and subjected to two-step FACS-aided purification. Control FACS profile on day 5 of cells derived from mES (J1) cells (A). Representative FACS profiles of day 5, with vascular progenies assessed using anti-Flk1 and anti-VE-cadherin antibodies obtained from mES (J1) cells (B) and derived from miPS (iMZ-21) cells (C); Control FACS profile on day 5 of cells derived from miPS (iMZ-21) cells (D). Representative FACS after the second step of purification derived from mES (E) and iPS cells (F). The yield of Flk1^+^VE-cadherin^+^ after the second round of FACS was 100% for both mES and miPS-derived vascular progenies. Morphology of mES- and miPS-derived vascular ECs (G-J). Flk1^+^VE-cadherin^+^ vascular progenies derived from mES and miPS cells were cultured overnight in IV Col-coated dishes, immunostained with anti-VE-cadherin (green) and anti-CD31 (red) of cells derived from mES cells (G&H) and miPS cells (I&J). DAPI, nucleus (blue). Magnifications are as indicated; the scale bar is 200 µm. Experiments were repeated 3 times.

After establishing day 5 as the optimal time for generation of Flk1^+^ VE-cadherin^+^ cells from both miPS and mES cells, we used this time point for all subsequent studies. We subjected the day 5 cells to a 2-step purification procedure using anti-Flk1 and anti-VE-cadherin antibodies to sort Flk1 and VE-cadherin double positive cells (see Methods for details) (Figure 2B&E shows cells derived from J1 line and Figure 2C&F shows cells derived from iMZ-21 line). In the first purification step, sorting of mES cells gave rise to 40±9% Flk1^+^ and VE-cadherin^+^ cells (Figure 2B), whereas miPS cells yielded 35±10% Flk1^+^ and VE-cadherin^+^ cells (Figure 2C). Upon subjecting these cells to a second round of purification, we obtained nearly 100% Flk1^+^ and VE-cadherin^+^ cells (Figure 2E, from mES; Figure 2F from miPS). Further analysis of the gated Flk1^+^ and VE-cadherin^+^ cells derived from mES and miPS cells showed that they lacked CD14 and CD45 (data not shown). In addition, anti-VE-cadherin and anti-CD31 antibodies staining confirmed the EC nature of the day 5 Flk1^+^VE-cadherin^+^ cells derived from mES cells (Figure 2G&H) as well as miPS cells (Figure 2I&J).

### mRNA expression profile of mES and miPS cell-derived ECs

**Figure 3:**
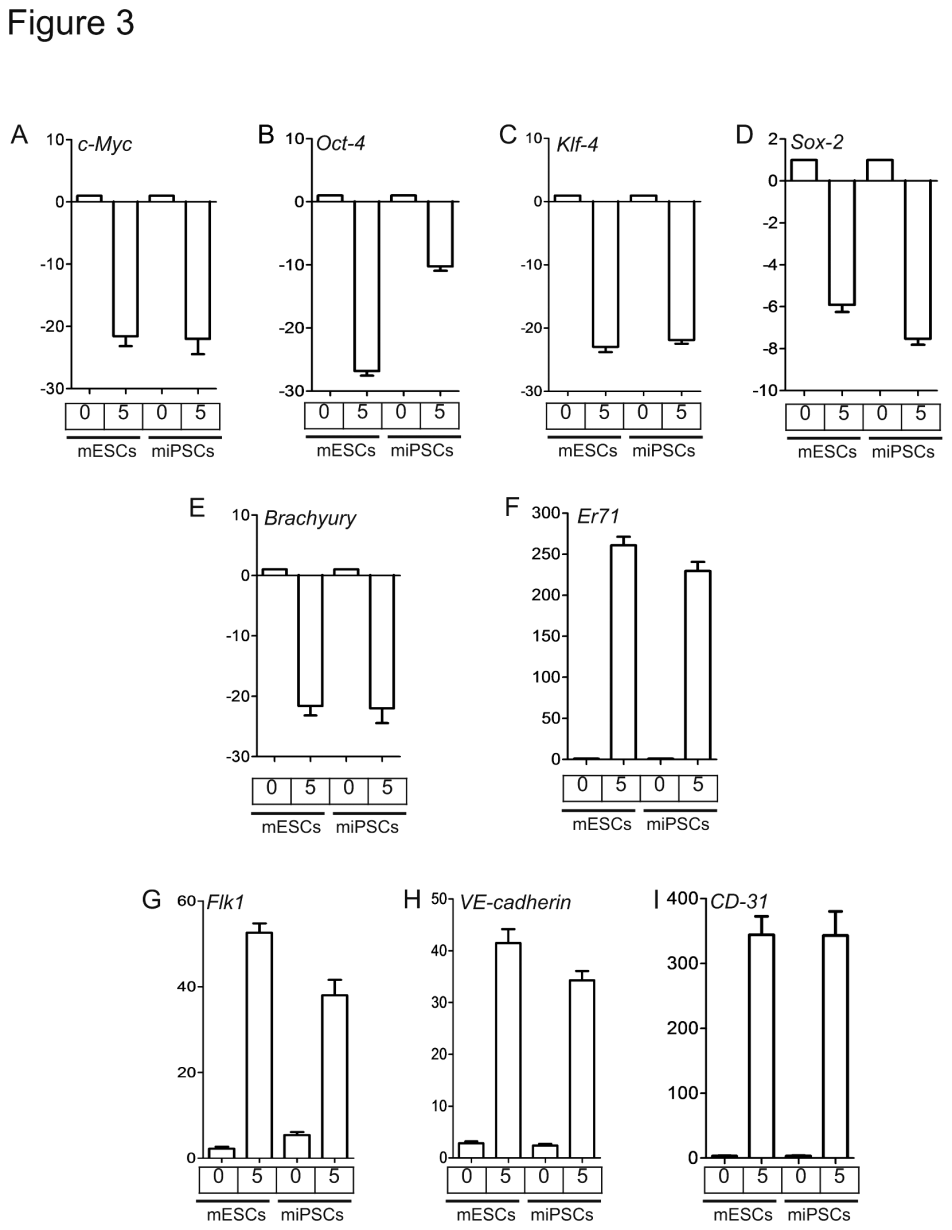
Gene expression profiles of ECs derived from mES and miPS cells. Total mRNAs were prepared from mES and miPS cells at day 0 and FACS-sorted Flk1^+^VE-cadherin^+^ ECs at day 5, subjected to q-RT-PCR analysis thereafter as described in methods. Expression of transcription factors c-Myc, Oct4, Klf4, and Sox2 in mES and miPS on indicated days (A-D). Decreased expression of Brachyury and but increased Er71 indicated development of mesodermal-endothelial progenies (E&F). Highly enriched expression of Flk1, V*E-cadherin*, and CD31 transcripts indicated acquisition of the EC phenotype (G-I). Data represent mean +S.E.M., n=5. Experiments were repeated at least 5 times with quadruplicates.

We next prepared mRNAs and mouse gene specific oligonucleotides primers (Table 1), and assessed by q-RT-PCR the expression of pluripotent stem cell markers *c-Myc*, *Klf4*, *Oct4*, and *Sox2*; the mesodermal/mesenchymal markers *Brachyury and Er71*; and mature EC markers *Flk1*, *VE-cadherin*, and *CD31* on days 0 and 5. Naive (undifferentiated) mES and iPS cells expressed *c-Myc*, *Klf4*, *Oct4*, *Sox2* on day 0, whereas expression decreased significantly by day 5 (Figure 3A-D). Because *Brachyury* and *Er71* are critical for mesodermal/EC fate [18,20,21,27,31,32], we examined their expression in mES- and miPS-cells and in ECs derived from mES- and miPS-cells (Figure 3E&F). Expression of the mesodermal marker *Brachyury* decreased on day 5 cells but *Er71* expression remained high on day 5 in cells obtained from both mES and miPS cells (Figure 3F). Importantly, the expression of the EC markers *Flk1*, *VE-cadherin* and *CD31* in both mES and miPS cells was low on day 0, but increased significantly by day 5 (Figure 3G-I). However, expression of the control *Gapdh* remained unaltered in all cells (data not shown). Expression of the hematopoietic cell markers *CD45* and *CD14* genes was low throughout (data not shown). 

### Er71 binds to *Flk1*-, VE-cadherin-, and CD31-promoters during differentiation to endothelial lineage

**Figure 4:**
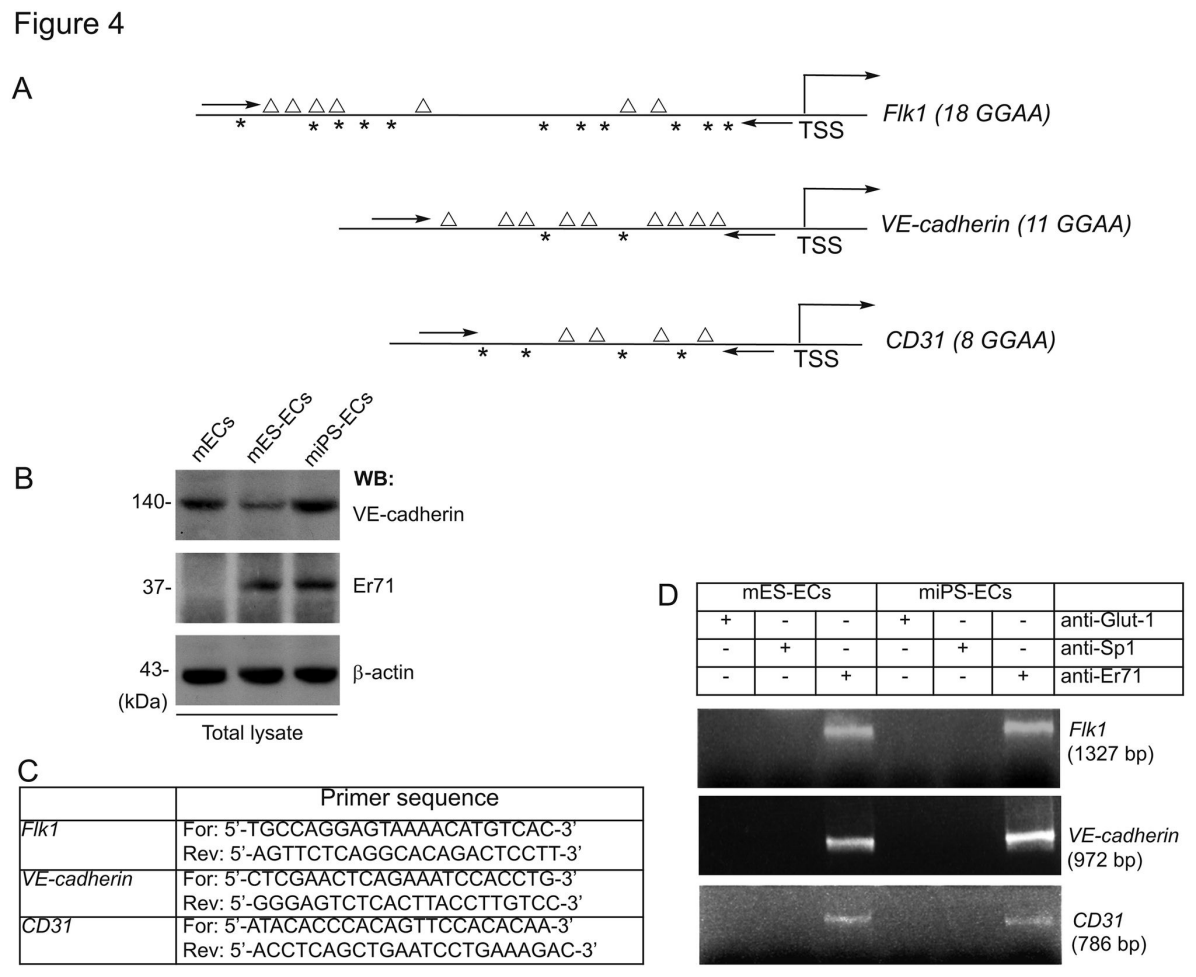
Er71 binds to *Flk1-, VE-cadherin-*, and CD31-promoters in mES and iPS cells. Schematics of promoter/enhancer regions of *Flk1*- (-1.4 kb), VE-cadherin- (-1.0 kb), and CD31- (-1.0 kb) harboring GGAA sites (triangles represent GGAA sites on the forward strand and asterisks represent these in the reverse strand), and the number within parentheses indicates the number of putative Er71 binding sites; TSS, transcription start site (A). Total lysates were subjected to Western blot analysis with indicated antibodies (B). The primers used for the amplification of the induced promoter/enhancer in the mouse genome (C). Flk1^+^VE-cadherin^+^ cells derived from mES- and miPS-cells were cultured overnight. ChIP was performed with antibodies (Abs) specific for Glut-1 (control), Sp1, and Er71. Representative images of PCR products from the mouse Flk1, VE-cadherin, and CD31 promoter/enhancer with the use of input chromatin prepared from indicated ECs. PCR products are as shown (D). The experiments were carried out 3 times.

We observed increased expression of the *Ets* transcription factor *Er71* (Figure 3F). Because Er71 regulates Flk1 and VE-cadherin expression in physiological vascular development [12,26-28], we examined whether Er71 was functional in Flk1^+^ and VE-cadherin^+^ ECs derived from mES- and miPS-cells. DNA sequences identified putative Er71 binding sites (*GGAA*) within the -0.7 kb to -1.4 kb upstream genomic segment of transcription starts sites (TSS) of *Flk1*, *VE-cadherin*, and *CD31* promoter DNA sequences (Figure 4A; Figures S1-S3). We also observed the expression of Er71 and VE-cadherin proteins by Western blotting (Figure 4B). We performed the ChIP assay to identify sites bound by Er71 in the promoters of Flk1, CD31, and VE-cadherin of ECs obtained from either mES or iPS cells. Immunoprecipitation studies were carried out using anti-Glut-1 (control), anti-Sp1, and anti-Er71 antibodies. To analyze protein-DNA complex formation, we designed specific PCR primers (Figure 4C). Using these primers, we did not detect any binding of Glut-1 or Sp1 to *Flk1*, *VE-cadherin*, or *CD31* DNA sequences (Figure 4D). By contrast, Er71 bound similarly to *Flk1*, *VE-cadherin*, and *CD31* promoter sequences in ECs obtained from both mES and iPS cells. As Sp-1 constitutively occupies GGAA sites in the genome, we included anti-Sp1 antibody as an additional control for the ChIP experiments. ChIP analysis was shown to be specific for Er71 because *Flk1*, *VE-cadherin*, and *CD31* promoters were not detected using the anti-Glut-1 and anti-Sp1 antibodies (Figure 4D). 

### Flk1^+^VE-cadherin^+^ ECs derived from mES- and miPS-cells form vascular networks

**Figure 5:**
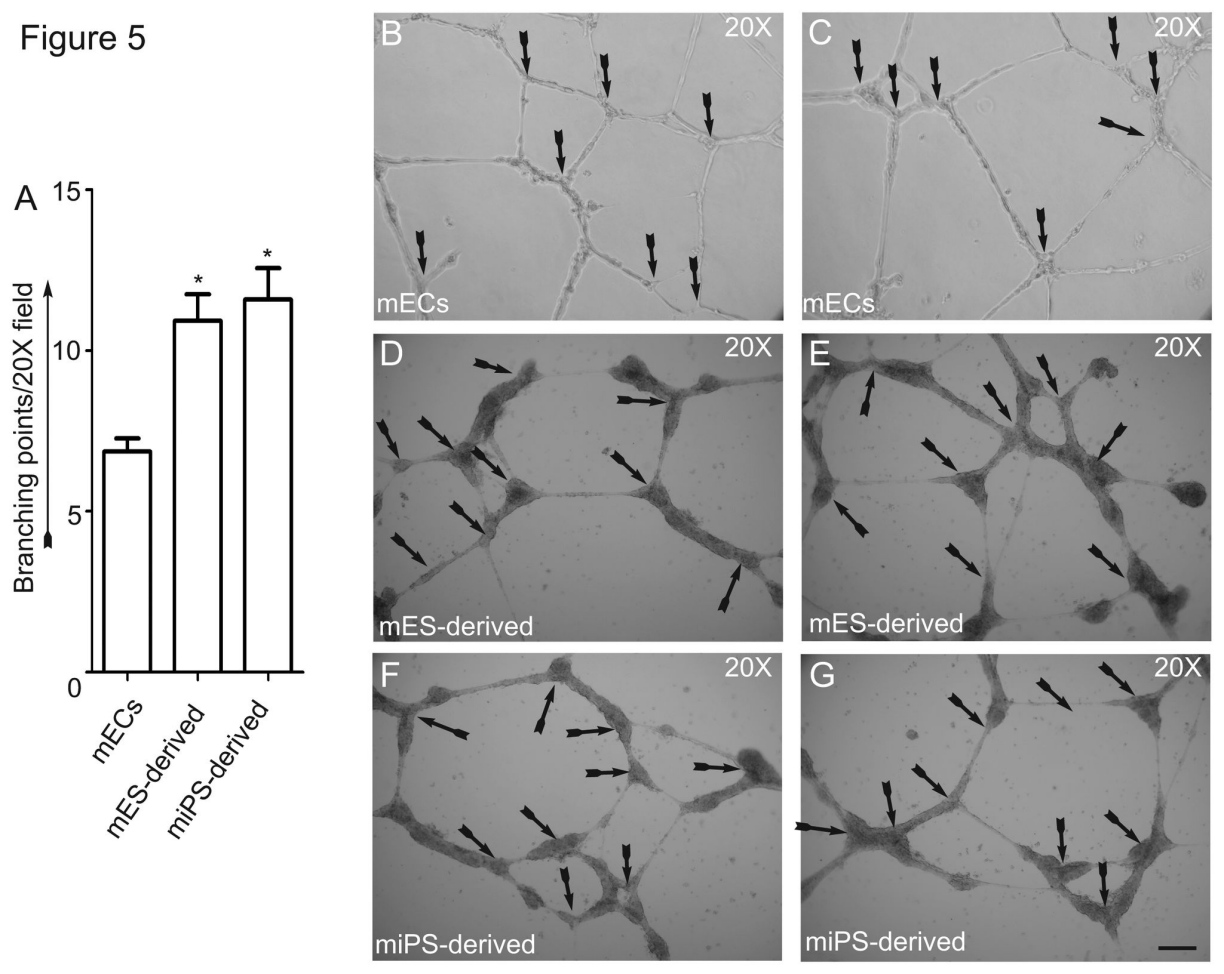
Angiogenic potential of Flk1^+^VE-cadherin^+^ ECs derived from mES and iPS cells. Quantification of branching point structures; control mouse ECs and mES- or iPS-derived Flk1^+^VE-cadherin^+^ ECs (2x10^5^) were plated onto Matrigel in the presence of VEGF^165^ (50 ng/ml). After 18 hr, the numbers of branching points were counted. Data are expressed as percentage of branching points (n=3, *P <0.05 vs. control or as indicated) (A); Representative phase contrast images of branching point structures. The experiments were repeated 3 times with the use of triplicate wells. Scale bar, 200 µm. The arrows indicate branching points (B-G).

ECs plated onto Matrigel *in vitro* are induced to undergo a morphogenic differentiation into vascular-plexus like networks or branching point structures, a process requiring cell adhesion and growth factor stimulation. To determine the neovascularization potential of the Flk1^+^ VE-cadherin^+^ cells, we first seeded the cells onto growth factor-reduced (GFR) Matrigel *in vitro* and quantified formation of branching points (Figure 5A). Plating of mature Flk1^+^VE-cadherin^+^ ECs (control) onto Matrigel-coated dishes resulted spontaneous assembly of branching point structures within 18 hr (Figure 5B&C). Plating of Flk1^+^VE-cadherin^+^ cells derived from either mES or miPS cells on growth factor-reduced Matrigel resulted in similar formation of branching point networks (Figure 5D-G). In the absence of VEGF, however, Flk1^+^VE-cadherin^+^ ECs failed to form interconnecting branching point networks (data not shown). Flk1^-^VE-cadherin^-^ cells did not form branching point structures (data not shown). The Flk1^+^VE-cadherin^+^ cells derived from mES and miPS cells both functioned similarly and responded to VEGF to form branching point structures. 

### Mouse ES or iPS cell-derived Flk-1^+^VE-cadherin^+^ cells incorporate into CD31^+^ vessels *in situ*

**Figure 6:**
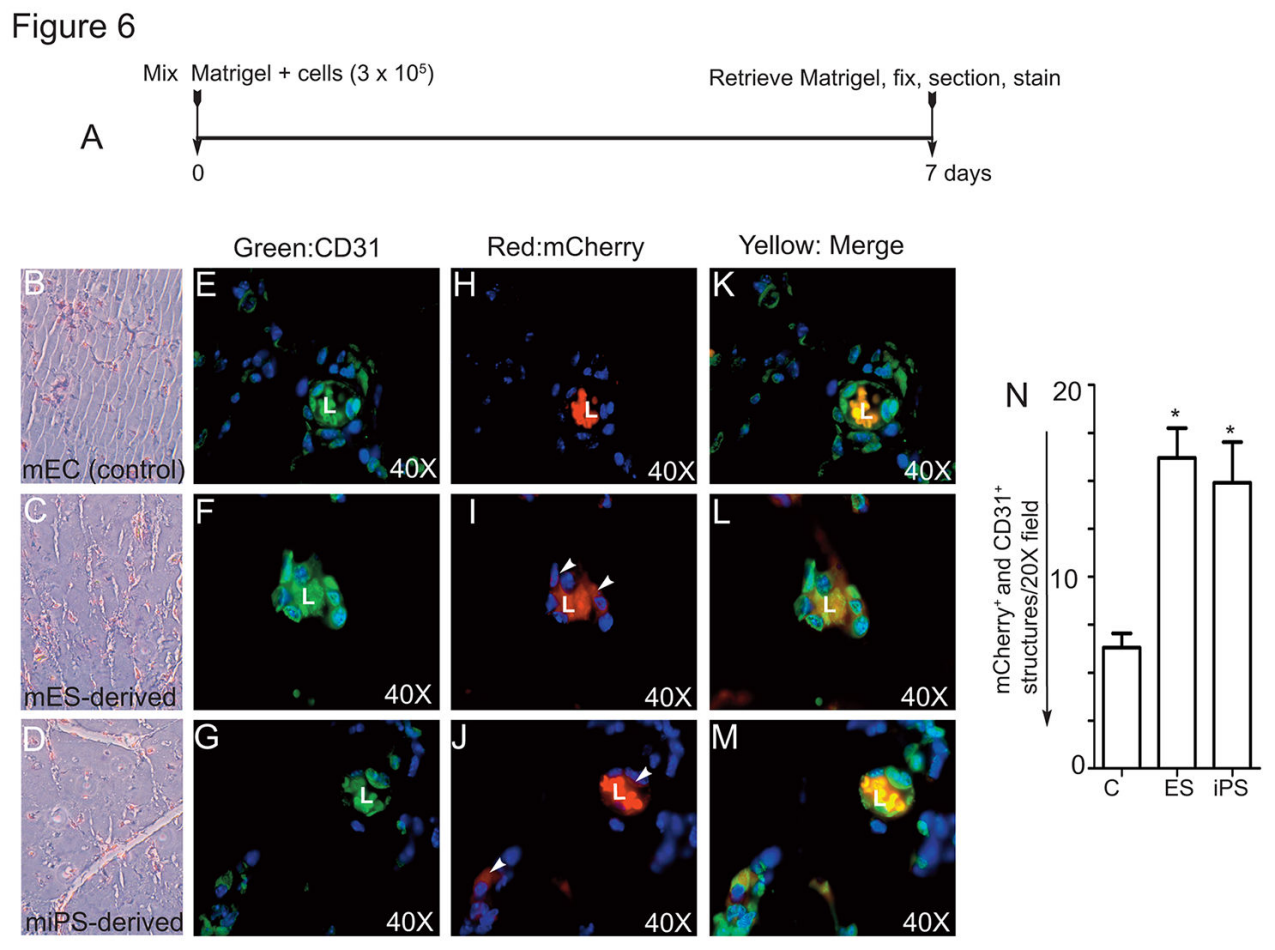
Mouse iPS-derived Flk1^+^VE-cadherin^+^ cells incorporate into CD31-positive vessels. Time line of the Matrigel experiment (A). Representative images of H&E stained Matrigel plugs obtained from nude mice receiving control mouse ECs or mES- or iPS-derived Flk1^+^VE-cadherin^+^ ECs (2x10^5^) (see Methods for details) (B,C,D). Immunohistochemistry of indicating that Matrigel sections were stained with anti-CD31 (green) and mCherry (red, arrowheads) (E-M). Autofluorescent leukocytes indicated perfused vessels (L). Quantification of number of neovessels in Matrigel sections incorporating the Flk1^+^VE-cadherin^+^ cells expressing mCherry (N). Scale bar 100 µm. Experiments were repeated at least 3 times.

To address whether mES- and miPS-derived Flk-1^+^VE-cadherin^+^ cells incorporated into vessels, we carried out the Matrigel plug assay using athymic nude mice. Both mES and miPS cells were differentiated as described above, and Flk1^+^VE-cadherin^+^ cells were sorted by FACS as described for Figure 1&2. The timeline of the experiment is shown (Figure 6A). Prior to mixing with the Matrigel, Flk-1^+^VE-cadherin^+^ cells were incubated overnight with lentiviral particles encoding the *mCherry* gene. We did not observe any detachment of cells or cell death after lentivirus-*mCherry* transfection of these cells, the morphology of lentivirus-transfected cells appeared normal, and transfection efficiency of mCherry was 100% (Figure S4). Matrigel was prepared and mixed with mCherry-expressing cells, and then injected subcutaneously. Matrigel plugs became visibly vascularized within 3-4 d. After 7 d, Matrigel plugs were collected and fixed, and formation of vascular structures was analyzed by H&E and anti-mouse CD31 staining (Figure 6B-D & Figure S5). Quantification demonstrated that Flk1^+^VE-cadherin^+^ cells obtained from mES and miPS cells were similar in their ability to incorporate into CD31^+^ neovessels, and did so in higher numbers compared with mature mECs (Figure 6N). Representative micrographs of immunofluorescent histochemistry are as shown (Figure 6E-K). Staining of Matrigel sections with anti-mouse CD31 (green) showed neovessels containing both donor (Flk1^+^VE-cadherin^+^, mCherry, red) and host cells (Figure 6E-M). 

**Figure 7:**
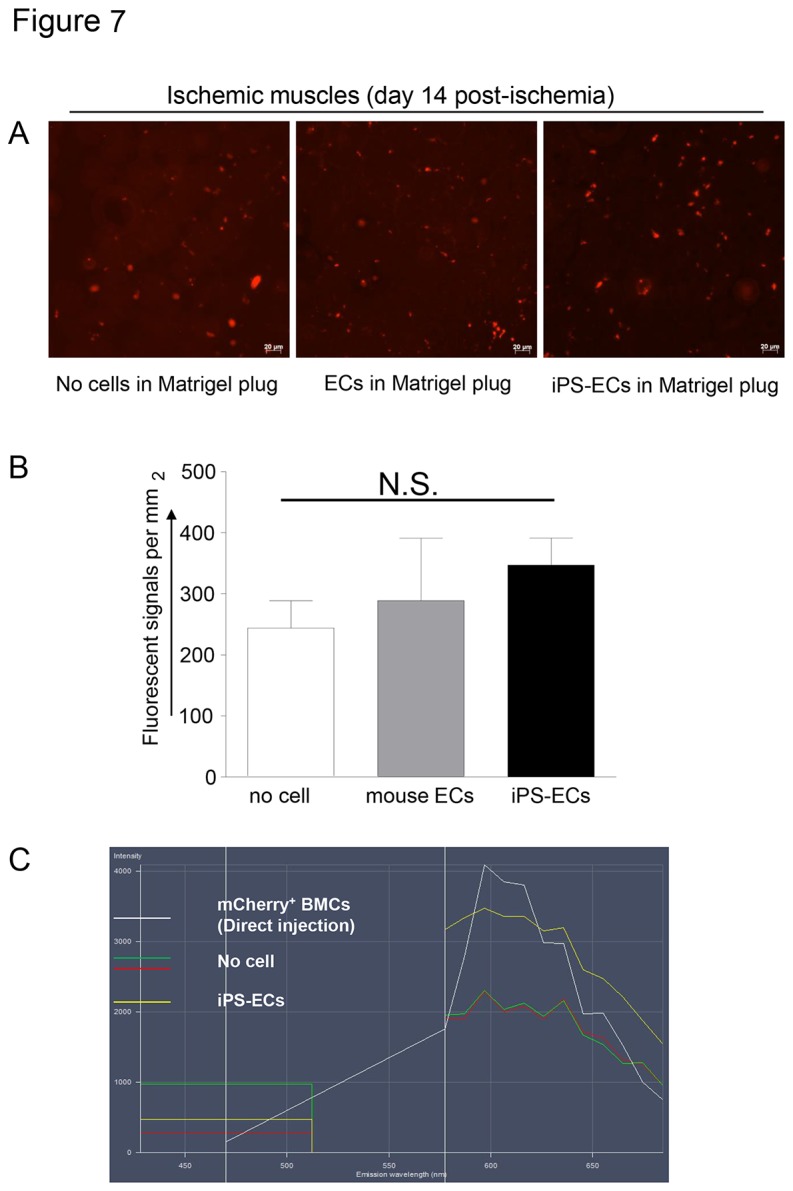
miPS derived ECs in the Matrigel plug are not mobilized to subsequent areas of injury. Mice were subjected to hindlimb ischemia 7 days after injection of Matrigel containing either no cells, mouse ECs or iPS cell-derived ECs. Cells were transduced with mCherry lentivirus particles before mixing with Matrigel. Ischemic tibialis anterior (TA) muscles were harvested 14 days after the surgery (21 days after Matrigel implantation). Cross sections of frozen tissues were observed under laser confocal microscopy. Images acquired with appropriate filter set for mCherry detection show fluorescent signals (20X objective) (A). Quantification demonstrated no appreciable difference in the numbers of fluorescent cells between the groups, suggesting that the obtained signals were likely due to autofluorescence and not due to a mobilization of the Matrigel-engrafted iPS-ECs (B). Fluorescence spectrum images were acquired by excitation with 405 and 562 nm and detection wave length with 470 nm and from 577 to 684 by 9-10 nm steps. The spectral analysis confirmed that the fluorescent signals from the hindlimbs of mice containing Matrigel plugs with mCherry labeled cells and those containing no cells were overlapping. Therefore, the fluorescent signals seen in (A) were indicative of autofluorescence of endogenous cells and did not demonstrate mobilization of iPS-ECs into the ischemic TA muscle (C). Experiments were repeated 3 times.

Since ischemia is known to mobilize adult progenitor cells [46], we monitored whether the Matrigel-engrafted iPSC-ECs would be mobilized by an ischemic insult. Mice were subjected to hindlimb ischemia 7 days after implantation of Matrigel containing either no cells, mouse ECs or iPS cell-derived Flk1+VE-cadherin+ ECs. To enable tracking of the cells, ECs were transduced with mCherry lentivirus particles before mixing with Matrigel. All animals survived the surgery and appeared to be healthy during the initial post-operative monitoring period. However, we did not find any appreciable number of mCherry positive cells in the ischemic TA muscle beyond the autofluorescence signal that was also seen in mice that did not receive any cells (Figure 7). These data collectively suggest that the Matrigel-engrafted cells did not mobilize to areas of injury, whereas iPS-ECs showed long-term stability ability by incorporating into CD31^+^ vessels.

## Discussion

Here we addressed the capacity of mES *vis-á-vis* miPS cells to produce Flk1^+^ and VE-cadherin^+^ EC lineage and compared the ability of both cell types as well as adult mouse ECs to induce vascularization. We showed that miPS-derived cells gave rise to Flk1^+^ and VE-cadherin^+^ ECs in a manner similar to ECs derived from mES cells. The temporal gene expression pattern associated with miPS differentiation led to expression of mesodermal- and endothelial-specific transcription factors followed by expression of mature EC proteins. This differentiating microenvironment was not limited only to the emergence of ECs *in vitro*, but also to the formation of functional and stable vessels *in vivo*. 

We used BMP4, VEGF, and bFGF to induce differentiation of iPS and ES cells utilizing IV Col as the supporting matrix protein [19,35]. Initially, we selected the iMZ-9 and iMZ-21 iPS cell lines, both of which were established by retroviral-mediated reprogramming of mouse embryonic fibroblasts with *Oct4*, *Sox2*, *Klf4* and *c-Myc* [4,5]. iMZ-9 and iMZ-21 iPS cells are equally capable of generating viable adult mice [4], therefore, pilot experiments were made side by side to compare the ability of both iPS cell lines to differentiate into ECs. For the purpose of the present experiments, we selected iMZ-21 cells because they consistently yielded a greater numbers ECs. We chose Flk1 as a marker for the EC phenotype because Flk1^+^ mesodermal cells are known to give rise to ECs *in vitro* and *in vivo* [14,17,19,35]. Flk1^+^ is also essential for EC identity and formation of a functional vasculature in culture and during embryonic development [12,14,15,17]. Flk1^+^ cells derived from mES or iPS cells, however, may represent a broader spectrum of mesodermal precursors than those giving rise only to ECs or hematopoietic cells. Therefore, we also selected VE-cadherin as a second marker for isolating and purifying ECs, since VE-cadherin is specifically expressed on ECs and essential for the maintenance of endothelial barrier function. Although VE-cadherin is considered a marker of mature ECs [24,25], we observed emergence of VE-cadherin^+^ cells by FACS as early as day 3, and the expression persisted until day 5 and beyond. These findings are significant as they show the potential to obtain similar EC differentiation from mES and miPS cells even though the iPS cells were generated using transcriptional factors and viral vectors. 

The temporal expression pattern of genes associated with the emergence of Flk1^+^ and VE-cadherin^+^ ECs mirrored that observed in the mouse embryonic vascular development [24-32]. Exposure of mES or iPS cells to the differentiation media for 5 days decreased expression of pluripotent stem cell markers (c-Myc, Klf4, Oct4 and Sox2) which was coupled with decreased expression of primitive streak and mesoderm markers (*Brachyury* and *Er71*). We observed concomitantly increased expression of hemangioblast/angioblast-specific transcription factors in both mES and miPS cells derived cells. iPS- and ES-derived Flk1^+^ and VE-cadherin^+^ cells however continued to express *Er71* even at the time when the cells showed expression of EC-specific Flk1, VE-cadherin, and CD31 cell surface proteins. *Er71* activates the transcriptional program specific to the endothelium, such as *Flk1, CD31*, and *VE-cadherin*, and these EC-specific genes are under the transcriptional control of Er71 [29,32]. Thus, the continued expression of Er71 may reflect the requirement of this factor to mediate the differentiation of ECs. These data together show that Flk1^+^ and VE-cadherin^+^ cells obtained from mES and miPS cells were not terminally differentiated into fully mature ECs. Importantly this finding helps explain the effectiveness of these cells in integrating into CD31^+^ vessels *in situ*. For the purpose of vascular tissue engineering, it is necessary that iPS derived endothelial cells remain integrated in a newly generated vessel and do not relocate to sites of ischemic injury. We subjected mice to an ischemic injury after implantation of a gel containing iPS-ECs and did not find any evidence of iPS-EC mobilization into ischemic tissues, thus indicating that the generated neovessels retain their stability even in the face of repeated injury.

In summary, we demonstrated that miPS cells produced Flk-1^+^ and VE-cadherin^+^ vascular EC progenies, expressed EC lineage transcription factors, and incorporated as ECs into CD31^+^ vessels *in vitro* and *in vivo*. This property of miPS cells was identical to mES cell-derived ECs and superior to that of adult mECs. Moreover, the emergence of Flk-1^+^ and VE-cadherin^+^ cells from miPS cells recapitulated the key steps of EC generation occurring during mouse embryonic development [26-28]. The ability to produce a large number of functional ECs from iPS cells and their engraftment potential therefore are likely to be significant value in vascular re-perfusion in ischemic tissue. 

## Supporting Information

Figure S1 **Mus musculus *Flk1*-promoter/enhancer -1.37 kb upstream of TSS.** Er71 binding site on (+) strand is shown in bold (GGAA) on the (-) strand bold underlined (TTCC).
(DOC)

Figure S2 **Mus musculus *VE-cadherin-*promoter/enhancer -1.03 kb upstream of TSS.** Er71 binding site on (+) strand is shown in bold (**GGAA**) on the (-) strand bold underlined (**TTCC**).
(DOC)

Figure S3 **Mus musculus *CD31-*promoter/enhancer -0.85 kb upstream of TSS.** Er71 binding site on (+) strand is shown in bold (**GGAA**) on the (-) strand bold underlined (**TTCC**).
(DOC)

Figure S4 **Morphology of Flk1^+^VE-cadherin^+^ mCherry expressing cells.** Flk1^+^ VE-cadherin^+^ cells derived from indicated cell lines were transduced with lentivirus encoding *mCherry* gene, under fluorescent microscope mCherry polypeptide appears bright red (this is due incorrect filter). The efficiency of transfection is 100%. Some of the cells appeared brighter than the others, perhaps due to different levels of expression and metabolic states. However, there was no toxicity or cell death associated with mCherry-lentivirus infection of the Flk1^+^VE-cadherin^+^ cells.
(TIF)

Figure S5 **Formation of neovessels in Matrigel plugs.** Matrigel plugs collected from nude mice were fixed, sectioned, and stained with H&E. (A&B) Flk1^-^ and VE-cadherin^-^ cells did not form functional neovessels. (C&D) Flk1^+^VE-cadherin^+^ cells derived from iPS and ES cells formed robust neovessels which were filled with leukocytes. Original magnifications are as shown. Scale bar, 200 µm.
(TIF)
