# Correction: Flk1+ and VE-Cadherin+ Endothelial Cells Derived from iPSCs Recapitulates Vascular Development during Differentiation and Display Similar Angiogenic Potential as ESC-Derived Cells

**DOI:** 10.1371/annotation/7c0448d9-bd2c-4367-957e-779dad721c9b

**Published:** 2014-01-29

**Authors:** Erin E. Kohler, Kishore K. Wary, Fei Li, Ishita Chatterjee, Norifumi Urao, Peter T. Toth, Masuko Ushio-Fukai, Jalees Rehman, Changwon Park, Asrar B. Malik

There are some missing reverse primers in the online version of Table 1. The PDF version of Table 1 is correct. Please refer to the PDF version for the correct Table 1.

