# Supplementary material for: Flk1+ and VE-Cadherin+ Endothelial Cells Derived from iPSCs Recapitulates Vascular Development during Differentiation and Display Similar Angiogenic Potential as ESC-Derived Cells
Source: PLoS One. 2013 Dec 30;8(12):e85549. doi: 10.1371/journal.pone.0085549 (PMC3875577; doi:10.1371/journal.pone.0085549)
Supplement: Figure S1 — Mus musculus Flk1-promoter/enhancer -1.37 kb upstream of TSS. Er71 binding site on (+) strand is shown in bold (GGAA) on the (-) strand bold underlined (TTCC). (DOC) [file pone.0085549.s001.doc]

**Figure S1.** *Mus musculus Flk1*-promoter/enhancer.

1 ATCATTCTAA ATGCATTTGG TTTTTGCCAG GAGTAAAACA TGTCACAAGA TATTTGTTGT

61 CAT**TTCC**CAG GCGT**GGAAGG** **AA**AGGAAT**GG** **AA**AGAAAACG AGGGGTGAAG GCTGCTG**TTC**

121 **C**TCTCTAGTC GCTACTTGAA GTCTACATAG CTGGGGGGGG GGGGGGGACT GTTCACATGG

181 GACCGGT**TTC** **C**TCTTTG**TTC** **C**TACACTGGC GCCTCTGGCA AGAAACTCTC CCTTCTC**TTC**

241 **C**CCCCAAGCA TATCTTGGCT GAAAGGTCAG CTCTGAAAAG GGGCCTGGCC AAAGTTACTG

301 TAGGGGACCG TGGTCAT**GGA** **A**CTGGGTAGA CAAAAGCACT CTAGCAGCCA CTGGAGAAGG

361 ACCGGGGGCT CTTCTCTGTG CATTTGCCCT GGAGCCCTGA CCACCGCCAG CTCCCTGCAT

421 CTCCTTGCTA TGGGTTTTCT GGACCGAGCC AGGCAGGAGT TCACAACCGA AATGTCTTCT

481 AGGGCTAATC AGGTAACTTC GGACGATTTA AAGTTGCCAG ATGGACGAGA AAACAGTAGA

541 GGCGTTGGCA ACCTGGATAA GCGCCTATCT TCTAATTAAA ACATTCAGAC GGGGCGGGGG

601 ATGCGGTGGC CAAAGCACCA TAAAACAAAA C**TTCC**AAGTA CTGACCAACT CACTGCAAGT

661 TTGTGCCCCG AGTACATCTA GGTTCAGGGG TTCTTGTCTT CATGCTCCCA ACTGCGGGCG

721 GATTTTTGGT CCCTTGGGAC TTTCAGTGCA GCGGCGAAGA GAGTTCTGCA CTTGCAGGCT

781 CCTAATGAGG GCGCAGTGGG CCTCGTGTTT CTGGTGATGC **TTCC**CAGGTT GCTGGGGGCA

841 GCAAGTGTCT CAGAGCCCAT TACTGGCTAC ATTTTAC**TTC** **C**ACCAGAAAC CGAGCTGCGT

901 CCAGATTTGC TCTCAGATGC GACTTGCCGC CCGGCACAG**T** **TCC**GGGGTAG TGGGGGAGTG

961 GGCGTG**GGAA** ACCG**GGAA**AC CCAAACCTGG TATCCAGTGG GGGGCGTGGC CGGACGCAGG

1021 GAGTCCCCAC CCCTCCCGGT AATGACCCCG CCCCCATTCG CTAGTGTGTA GCCGGCGCTC

1081 TCTTTCTGCC CTGAGTCCTC AGGACCCCAA GAGAGTAAGC TGTGT**TTCC**T TAGATCGCGC

1141 GGACCGCTAC CCGGCAGGAC TGAAAGCCCA GACTGTGTCC CGCAGCCGGG ATAACCTGGC

1201 TGACCCGA**TT** **CC**GCGGACAC CGCTGCAGCC GCGGCTGGAG CCAGGGCGCC GGTGCCCCGC

1261 GCTCTCCCCG GTCTTGCGCT GCGGGGGCGC ATACCGCCTC TGTGACTTCT TTGCGGGCCA

1321 GGGACGGAGA AGGAGTCTGT GCCTGAGAAC TGGGCTCTGT GCCCAGCGCG AGGTGCAGG
