# Supplementary material for: Flk1+ and VE-Cadherin+ Endothelial Cells Derived from iPSCs Recapitulates Vascular Development during Differentiation and Display Similar Angiogenic Potential as ESC-Derived Cells
Source: PLoS One. 2013 Dec 30;8(12):e85549. doi: 10.1371/journal.pone.0085549 (PMC3875577; doi:10.1371/journal.pone.0085549)
Supplement: Figure S2 — Mus musculus VE-cadherin-promoter/enhancer -1.03 kb upstream of TSS. Er71 binding site on (+) strand is shown in bold (GGAA) on the (-) strand bold underlined (TTCC). (DOC) [file pone.0085549.s002.doc]

**Figure S2.** *Mus musculus* VE-cadherin promoter/enhancer **(NC_000074.6)**

1 AGGGTTTCTC TGTATAGCCC TGGCTATCCT **GGAA**CACACT CTGTAGACCA GGCTGGCCTC

61 GAACTCAGAA ATCCACCTGC CTCTGCCTCC CGAGTGCTGG GATTAAAGGC GTGCGCCACC

121 ACGCCTGGCT GAATGAAGAG AATCTTGACC TCATCTCCCC AGCCTCTTGG TCCTGAGGGA

181 CCCTGGTCTA CCTACTGCTT TGCTGTCTTC TTAGCTCTTC TTACTTTTTT GCTGACTCAG

241 ACCTATGGCT ATCTCCATTA TACAGATGAG GAGACTGAGG CATGGATCCC TGGTTGGTCC

301 ATGGTCACGT GAAGCCCATC ACCCAGTATT TGTAAAGTGA GATGGGCCAG GCTGGTACCT

361 T**GGAA**CTGAA ACTCACACTG CCCTACCT**GG AA**GAATCTGA CAGGCAAAAT CTGCTGCTGA

421 AAGTGATTGT CTGTCACGTT TCTCAGCTGC CCGACTCTGA GAACTCCACA GCCCCCTTTC

481 G**TTCC**ACCAT ACTACAGAGT CGCCAC**GGAA** AGCCGGCTCT GTGGAGAAGC TGAGGTAGCT

541 GGGTTTCTGT CTGGGTTACT CTGTCCAGCG A**GGAA**ACAAG TACCTTAGAC CCACTAAGCC

601 TCTGCTTTCT GAACTGTAAA GTGGGGGATA TGACACCTGC CTCCCAGGGA TGGCTGAATG

661 CTCTGGCAGA AGCTTAGAGC CCCCACAGCT ACCCCTAGGC TCACAGCTCC TCCGATGAGA

721 CCTAGAATTG AGGTATGAGT TGAATACCCC AGGCAGGTCC AAGGC**TTCC**A CGGGCCCAGG

781 CTGACCAAGC TGAGGCCGCC CACCGTAGGG CTTGCCTATC TGCAGGCAGC TCACAAA**GGA**

841 **A**CAATAACA**G GAA**ACCATCC CGAGG**GGAA**G TGGGCCAGGG CCAGTT**GGAA** AACCTGCCTC

901 CCTCCCAGCC TGGGTGTGGC TCCCCTCTCC CCTCCTGAGG CAATCAACTG TGCTCTCCAC

961 AAAGCTCGGC CCTGGACAGA CTGCAGTGGA GAGAGCCTTC TGCTCACGGA CAAGGTAAGT

1021 GAGACTCCCA AATCCCTCTC TACT
