# Supplementary material for: Flk1+ and VE-Cadherin+ Endothelial Cells Derived from iPSCs Recapitulates Vascular Development during Differentiation and Display Similar Angiogenic Potential as ESC-Derived Cells
Source: PLoS One. 2013 Dec 30;8(12):e85549. doi: 10.1371/journal.pone.0085549 (PMC3875577; doi:10.1371/journal.pone.0085549)
Supplement: Figure S3 — Mus musculus CD31-promoter/enhancer -0.85 kb upstream of TSS. Er71 binding site on (+) strand is shown in bold (GGAA) on the (-) strand bold underlined (TTCC). (DOC) [file pone.0085549.s003.doc]

**Figure S3.** *Mus musculus* *CD31*-promoter/enhancer.

1 TGTGTACTAC ACACACATAT ATACCCACAG TCACAGACAC ACACATACAC CCACAG**TTCC**

61 ACACAAACAC ATATACACCC AGTCACACAC ACACACAAAC ACACATACAT ACATACATAC

121 ACAGTCACAC ACACACAAAC ACACACACAA ACACACATAC ACAAACAGCC AAAGGGGCTC

181 CTTTGGTGAC TAAGCCGGTG ATCAGGGACC ACTGCAGCGG CTTAAGAAAA TCCCTCTTTC

241 TACTGAGCCA CCAGTAGCG**T** **TCC**TCACAAG GACTGCATAT TGTCGGAGAA GCTGGCCAGT

301 GCAGGATAGG AGGCCCAGAT TTACACTCTA TGGATACAGC CCCCATAGGG AGCCCACA**GG**

361 **AA**AGGCAAAC AGGTCTTGCT AGGTATCCCA CAAAAGCACT TTTGAGT**GGA** **A**ATAAAACCT

421 CCTTGTCCTG TCAGGAGAAG AAGTCCCCAA ACCCAGATGA GGCTAGAAGC GCAAGAACTT

481 TGAGGCAAAT GCTACTTGTG GGTTTTTTTG GTTGTTGTTG TTGTTTTCTC CTCTTCTAGA

541 CAAAGCCATT GGGTTCAGTG GTCCCTCC**GG** **AA**GGCCAGTC AT**TTCC**TGAG GAGATATCAG

601 GCCAGCCCAG ACCTCATTGT TCTTGGT**TTC** **C**AGCTATGGC CGCCATTACC TGACAAGTAC

661 CAGAGCTGGT CTCTGCGCGC CTGGAGAGGT TGTCAGAGTT GTTTCTGCTT TTCACACAGC

721 AGGCTTCTGG TCTGTGGCAG GCAAGAGAAT TCTGCCCCTG AGGCATGGCC **GGAA**GACAGA

781 ACTAGCTGAG TGCTTCAGTC TGCAGAAGTC TTTCAGGATT CAGCTGAGGT GGGCCTCAGT

841 CGGCAGACAA G
