# Supplementary figures and images for: Flk1+ and VE-Cadherin+ Endothelial Cells Derived from iPSCs Recapitulates Vascular Development during Differentiation and Display Similar Angiogenic Potential as ESC-Derived Cells
Source: PLoS One. 2013 Dec 30;8(12):e85549. doi: 10.1371/journal.pone.0085549 (PMC3875577; doi:10.1371/journal.pone.0085549)

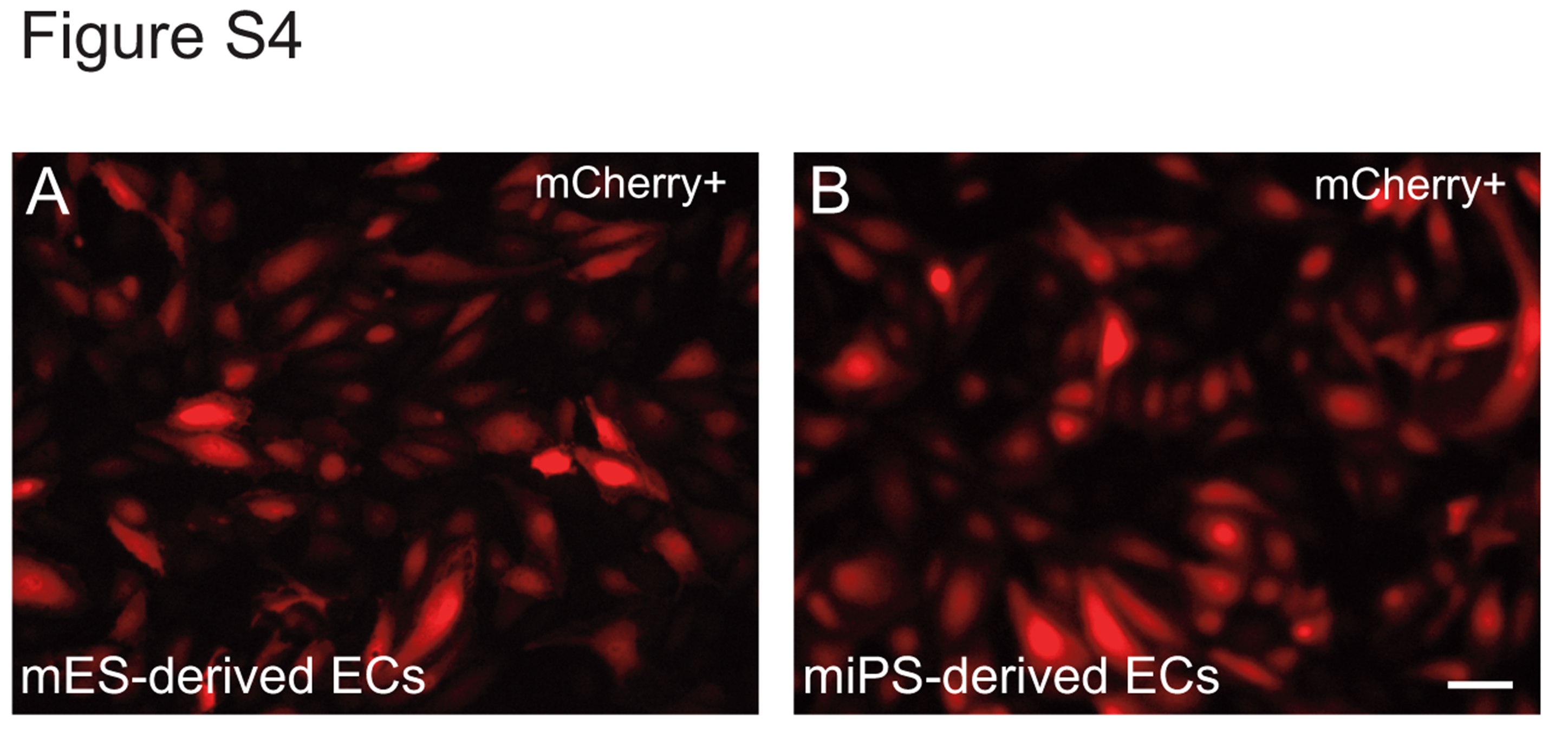

Supplement: Figure S4 — Morphology of Flk1+VE-cadherin+ mCherry expressing cells. Flk1+ VE-cadherin+ cells derived from indicated cell lines were transduced with lentivirus encoding mCherry gene, under fluorescent microscope mCherry polypeptide appears bright red (this is due incorrect filter). The efficiency of transfection is 100%. Some of the cells appeared brighter than the others, perhaps due to different levels of expression and metabolic states. However, there was no toxicity or cell death associated with mCherry-lentivirus infection of the Flk1+VE-cadherin+ cells. (TIF) [file pone.0085549.s004.tif]

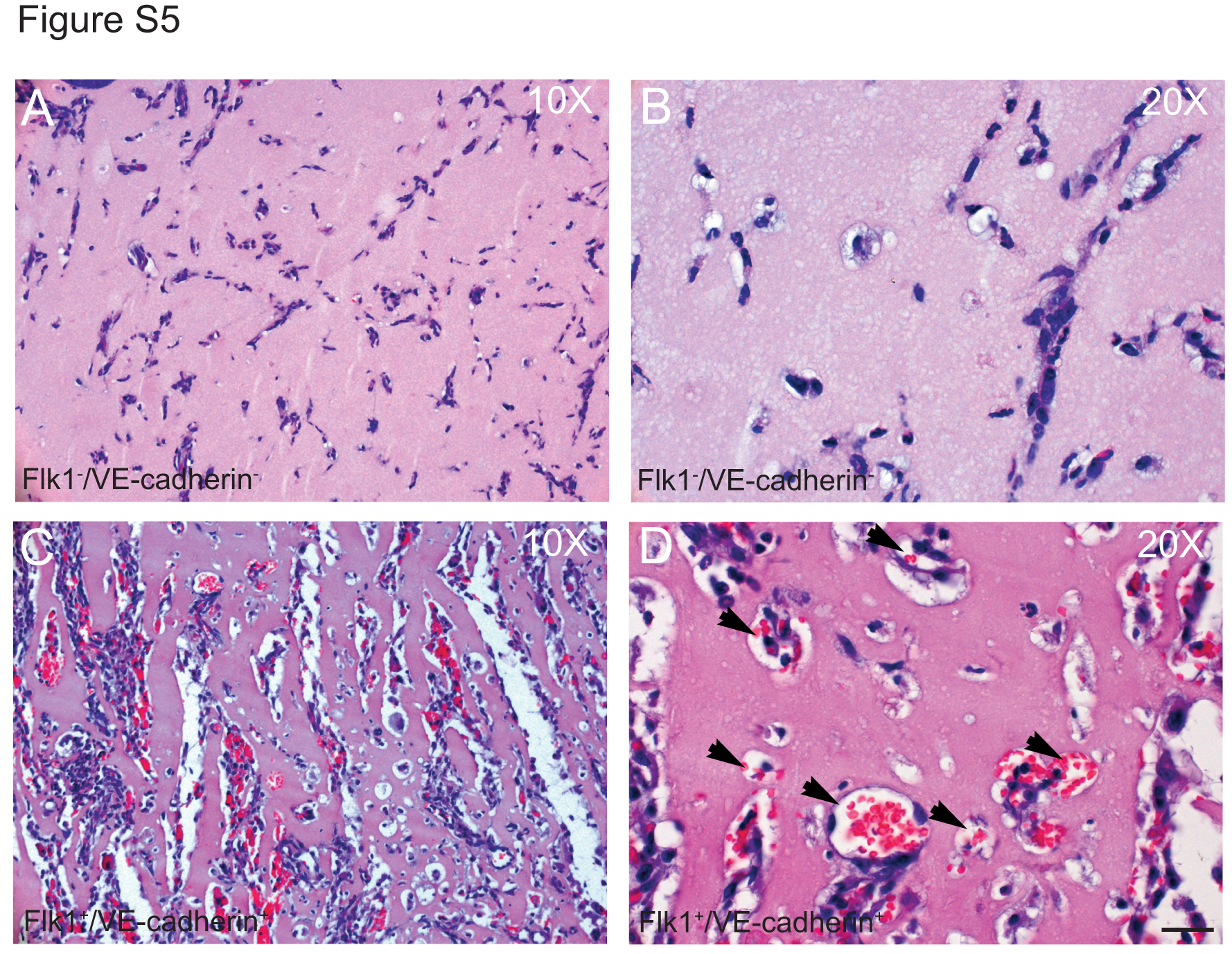

Supplement: Figure S5 — Formation of neovessels in Matrigel plugs. Matrigel plugs collected from nude mice were fixed, sectioned, and stained with H&E. (A&B) Flk1- and VE-cadherin- cells did not form functional neovessels. (C&D) Flk1+VE-cadherin+ cells derived from iPS and ES cells formed robust neovessels which were filled with leukocytes. Original magnifications are as shown. Scale bar, 200 µm. (TIF) [file pone.0085549.s005.tif]
